# Ovarian Cancer: Advances in Pathophysiology and Therapies

**DOI:** 10.3390/ijms24108930

**Published:** 2023-05-18

**Authors:** Giovanni Tossetta, Annalisa Inversetti

**Affiliations:** 1 Department of Experimental and Clinical Medicine, Università Politecnica delle Marche, 60126 Ancona, Italy; 2 Department of Biomedical Sciences, Humanitas University, 20072 Milan, Italy; 3 IRCCS Humanitas Research Hospital, Rozzano, 20089 Milan, Italy

We are pleased to present this Special Issue of the International Journal of Molecular Sciences, entitled “Ovarian Cancer: Advances in Pathophysiology and Therapies”.

Ovarian cancer is a gynecologic tumor with high mortality rate. The high lethality of this tumor is mainly due to the fact that it is often diagnosed at an advanced stage of disease [1]. Primary or acquired chemoresistance is a challenge for the management of most malignancies, including ovarian cancer [1,2,3,4,5]. The occurrence of chemoresistance significantly worsens the outcome of ovarian cancer. In fact, although platinum-based drugs (e.g., cisplatin, carboplatin and oxaliplatin) and paclitaxel show significant efficiency at the beginning of treatment, many patients relapse within 18 months due to the occurrence of chemoresistance [1,4].

One of the potential reasons for acquired chemoresistance in cancer cells is the enhanced antioxidant defense and the accumulation of DNA mutations, together with changes in epigenetics [1,6,7,8].

It has been reported that the majority of ovarian cancers are epithelial ovarian cancer (EOC), but different histological subtypes of EOC have been identified, including serous (the most common), endometrioid, clear cell and mucinous (very rare) EOC [1].

The evaluation of the expression of specific biomarkers (microRNAs, lncRNAs, proteins, etc.) has produced important results for predicting the occurrence/progression of several cancerous [9,10,11,12,13] and non-cancerous [14,15,16] diseases. Moreover, some biomarkers have shown the ability to predict recurrence, chemoresistance, progression and metastasis development in several types of cancers, including ovarian cancer [17,18,19,20,21]. Thus, the use of these biomarkers may play a significant role in clinical practice, allowing personalized therapies that can improve outcomes for patients with ovarian cancer.

Oxidative stress plays a key role in oncogenesis, promoting its initiation and progression. Moreover, oxidative stress can activate important transcription factors, including nuclear factor (NF)-kB, p53, hypoxia-inducible factor 1 (HIF-1), peroxisome proliferator-activated receptor γ (PPARγ), and nuclear factor erythroid 2-related factor 2 (NFE2L2 or NRF2), which can modulate the expression of several genes involved in important cellular processes, including inflammatory responses, apoptosis, cell proliferation and differentiation [1,4,5,8].

Natural compounds are present in plants (e.g., carotenoids, anthocyanins and flavonoids), bacteria, fungi and marine organisms, and have shown important antioxidant, anti-inflammatory and anticancer effects in many tumors [5,8,22,23]. In fact, these compounds can prevent carcinogenesis, thereby reducing oxidative stress. Moreover, they can attenuate chemotherapeutic resistance in cancer cells, restoring drug sensitivity. Finally, several natural compounds can regulate cytokine production in the tumor microenvironment, therein modulating cancer cells’ growth and proliferation [5,8,23].

Understanding the mechanisms involved in the regulation of ovarian cancer’s development and progression may lead to new perspectives on the treatment of this pathology, thereby improving its outcome.

Thus, the aim of this Special Issue is to provide an overview of the molecular and signaling alterations involved in ovarian cancer’s development, diagnosis and treatment.
